# An Accelerometry-Based Methodology for Assessment of Real-World Bilateral Upper Extremity Activity

**DOI:** 10.1371/journal.pone.0103135

**Published:** 2014-07-28

**Authors:** Ryan R. Bailey, Joseph W. Klaesner, Catherine E. Lang

**Affiliations:** 1 Program in Physical Therapy, Washington University School of Medicine in St. Louis, St. Louis, Missouri, United States of America; 2 Program in Physical Therapy, Mallinckrodt Institute of Radiology, Department of Biomedical Engineering, Washington University School of Medicine in St. Louis, St. Louis, Missouri, United States of America; 3 Program in Physical Therapy, Program in Occupational Therapy, Department of Neurology, Washington University School of Medicine in St. Louis, St. Louis, Missouri, United States of America; Delft University of Technology (TUDelft), Netherlands

## Abstract

**Background:**

The use of both upper extremities (UE) is necessary for the completion of many everyday tasks. Few clinical assessments measure the abilities of the UEs to work together; rather, they assess unilateral function and compare it between affected and unaffected UEs. Furthermore, clinical assessments are unable to measure function that occurs in the real-world, outside the clinic. This study examines the validity of an innovative approach to assess real-world bilateral UE activity using accelerometry.

**Methods:**

Seventy-four neurologically intact adults completed ten tasks (donning/doffing shoes, grooming, stacking boxes, cutting playdough, folding towels, writing, unilateral sorting, bilateral sorting, unilateral typing, and bilateral typing) while wearing accelerometers on both wrists. Two variables, the Bilateral Magnitude and Magnitude Ratio, were derived from accelerometry data to distinguish between high- and low-intensity tasks, and between bilateral and unilateral tasks. Estimated energy expenditure and time spent in simultaneous UE activity for each task were also calculated.

**Results:**

The Bilateral Magnitude distinguished between high- and low-intensity tasks, and the Magnitude Ratio distinguished between unilateral and bilateral UE tasks. The Bilateral Magnitude was strongly correlated with estimated energy expenditure (ρ = 0.74, p<0.02), and the Magnitude Ratio was strongly correlated with time spent in simultaneous UE activity (ρ = 0.93, p<0.01) across tasks.

**Conclusions:**

These results demonstrate face validity and construct validity of this methodology to quantify bilateral UE activity during the performance of everyday tasks performed in a laboratory setting, and can now be used to assess bilateral UE activity in real-world environments.

## Introduction

Upper extremity (UE) function is necessary for the performance of many everyday tasks. Some tasks are performed using symmetrical movements between the UEs where kinetic and kinematic parameters are matched (e.g. carrying a heavy object) [1]. Other tasks are performed unilaterally (e.g. typing with one hand). Most tasks, including many “unilateral” tasks, actually occur in between these two extremes. Classified as bilateral complimentary activity, these tasks require both extremities to work together to accomplish a goal even though one extremity may be “functionally inactive.” An example of this is writing, where one hand is used to stabilize a piece of paper while the other hand manipulates a pen to write on the paper. Because most everyday tasks are completed using bilateral actions, bilateral UE function should be assessed in patients with UE impairment receiving rehabilitation services.

Surprisingly, few clinical assessments measure bilateral UE function. Many assessments measure UE function of the impaired extremity and compare it to function of the unimpaired extremity (e.g. Action Research Arm Test, Jebsen-Taylor Hand Function Test) [2]. Some assessments use bilateral tasks to measure UE function. The Chedoke Arm and Hand Inventory [3], for example, measures the ability to use both UEs to complete a task, but scoring is determined by the amount of assistance required to complete the task rather than any inherent characteristic of motor ability (e.g. speed, intensity). A further limitation of clinical assessments is that they do not measure free-living or real-world UE activity, defined as use of the UEs *outside of the clinic* to complete functional and non-functional tasks. For practical reasons, a clinician cannot personally track the activity of a patient 24 hours a day. Self-report measures of physical activity may be used to overcome this barrier, but self-reported activity is known to vary greatly with direct measures of activity [4] for many reasons, including desire for social approval [5] and cognitive impairment [6]. Clearly, existing clinical assessments are insufficient for measuring real-world bilateral UE function following UE impairment.

In an effort to measure real-world UE function, accelerometry has been introduced as an objective method to quantify real-world UE activity in healthy [7] and patient [8] populations. While accelerometry cannot distinguish arm movements that are functional (e.g. getting dressed) from non-functional (e.g. arm swing while ambulating), they serve as a useful *index* of real-world UE function (i.e. UE activity) [9]. Accelerometry has been used to quantify duration and intensity of UE activity of individual extremities, as well as duration and intensity of one extremity relative to the other extremity. This approach is the same as that described for clinical assessment: unilateral activity of each UE is assessed separately and then compared. Unfortunately, UE activity of one extremity *relative* to the other extremity is not the same thing as bilateral UE activity.

As a result of these challenges, this study examined the validity of an innovative approach that uses accelerometry data to quantify bilateral UE activity during the performance of every-day tasks. Participants completed 10 everyday tasks while wearing accelerometers. Two variables were calculated from the accelerometry data, the Bilateral Magnitude and Magnitude Ratio, to reflect bilateral activity intensity and the contribution of each UE to activity. We hypothesized that these variables would distinguish high intensity tasks from low intensity tasks, and bilateral tasks from unilateral tasks. We also hypothesized that the variables would be associated with estimated energy expenditure and time spent in activity when both UEs were simultaneously active.

## Methods

### Participants

Participants for this cross-sectional study were recruited through HealthStreet, a community-based effort of the Institute of Clinical and Translational Sciences at Washington University in St. Louis between May and September 2012. Inclusion criteria were (a) age >30 years, (b) ability to follow commands, and (c) dwelling in the community. Exclusion criteria were (a) self-reported history of a neurological condition and (b) self-reported history of significant UE impairment.

### Ethics

This study was approved by the Human Research Protection Office of Washington University and conformed to the Declaration of Helsinki. A total of 74 adults provided written informed consent, participated in the study, and were compensated for their time.

### Procedure

Participants completed a one-hour office visit at the Neurorehabilitation Lab at Washington University School of Medicine in St. Louis, where they provided demographic information, including self-reported hand dominance. Accelerometry was used to measure UE activity during task performance. The validity and reliability of accelerometry to measure UE activity is well-established [8], [10], [11], [12], [13]. The GT3X+ Activity Monitor (Actigraph, Pensacola, FL) contains a solid state, digital accelerometer that is capable of measuring acceleration along three axes, contains 512 MB of internal storage, and has ±6 g dynamic range. Acceleration was sampled at 30 Hz. Two accelerometers (one on each UE) were placed on distal forearms, proximal to the styloid process of the ulna, which allowed both proximal (i.e. upper arm) and distal (i.e. forearm) movements to be captured. Small movements of the hands and fingers that occur in isolation of more proximal segments, as occurs when one types on a computer but rests the forearms on a table surface, may be missed by accelerometers worn at the wrists; thus, wrist-worn accelerometry may slightly *underestimate* the actual amount of UE activity that occurs during task performance.

Participants performed eight UE tasks. The tasks were chosen to encompass a variety of UE movement patterns, including unilateral activity, symmetrical bilateral activity (where temporal, kinetic, and kinematic parameters were similar between UEs), and complementary bilateral activity (where the UEs were used in an asymmetrical but cooperative fashion to complete a task), that might be performed in real-world environments [1]. Tasks included donning/doffing shoes, grooming, stacking boxes, cutting playdough, folding towels, writing, sorting items into a tackle box, and typing. Some participants completed typing and sorting tasks predominantly one-handed (i.e. unilateral), while others completed the tasks using both hands (i.e. bilateral), resulting in ten tasks that were analyzed. A brief description of each task is given in Table 1. Task order was randomized using a custom-written program in MATLAB R2011b (Mathworks, Natick, MA), and task performance was video-recorded.

**Table 1:** Description of UE Tasks.

Task	Description
Shoes	Donning and doffing shoes, including tying laces if applicable.
Grooming	Tasks requiring bilateral UE activity that occurs around the head (e.g. combing/styling hair, removing/replacing earrings, mimed make-up application, shaving in front of a mirror).
Boxes	Transferring boxes (0.91 kg; 24 cmx15 cmx9.5 cm) between shelves located at shoulder- and waist-heights.
Cutting	Cutting playdough on a cutting board using a knife and fork.
Towels	Folding large bath towels and placing them into a pile.
Writing	Writing a short story on a piece of paper using a pencil.
Unilateral Sorting	Sorting small objects into a tackle box *with one hand* using a 3 point pinch (3-jaw-chuck).
Bilateral Sorting	Sorting small objects into a tackle box *with both hands* using a 3 point pinch (3-jaw-chuck).
Unilateral Typing	Typing a short story on a laptop computer using *one hand*.
Bilateral Typing	Typing a short story on a laptop computer using *both hands*.

To approximate movement patterns that might occur during real-world activity, participants were instructed to complete each task in a self-selected manner until the task was completed, which took between one and two minutes. Because participants were allowed to complete tasks in a self-selected manner, participants performed Bilateral Typing and Bilateral Sorting using a variety of symmetrical and complementary actions. For example, some participants were skilled typists who used both hands to type in a symmetric manner, while others were less skilled and typed by using the index fingers of both hands in a hunt-and-peck fashion. For Bilateral Sorting, some participants sorted objects using both hands at the same time, while others sorted objects by either using one hand at a time or using one hand continuously and occasionally using the other hand to help.

Participants wore the accelerometers for the next 24 hours while they went about their normal, daily routine at home. Summary analysis of accelerometry data collected during the 24 hours is reported elsewhere [14] and is not provided within this manuscript. Accelerometers were returned to the Neurorehabilitation Lab at the conclusion of the wear period, where accelerometry data were downloaded to a computer using ActiLife 6 proprietary software (ActiGraph, Pensacola, FL). ActiLife 6 software band-pass filters acceleration data between frequencies of 0.25–2.5 Hz, removes the effect of gravity, down-samples 30 Hz data into one second intervals by summing acceleration across samples, and converts acceleration into units called Activity Counts (1 Activity Count = 0.001664 g = 0.0163 m*s^−2^) [15]. Activity Counts for each task and each participant can be found in an online data repository at http://digitalcommons.wustl.edu/open_access_pubs/2901/. ActiLife 6 was also used to visually inspect accelerometry data to ensure that the accelerometers functioned properly during the recording period.

### Variables of Interest

**Figure 1:**
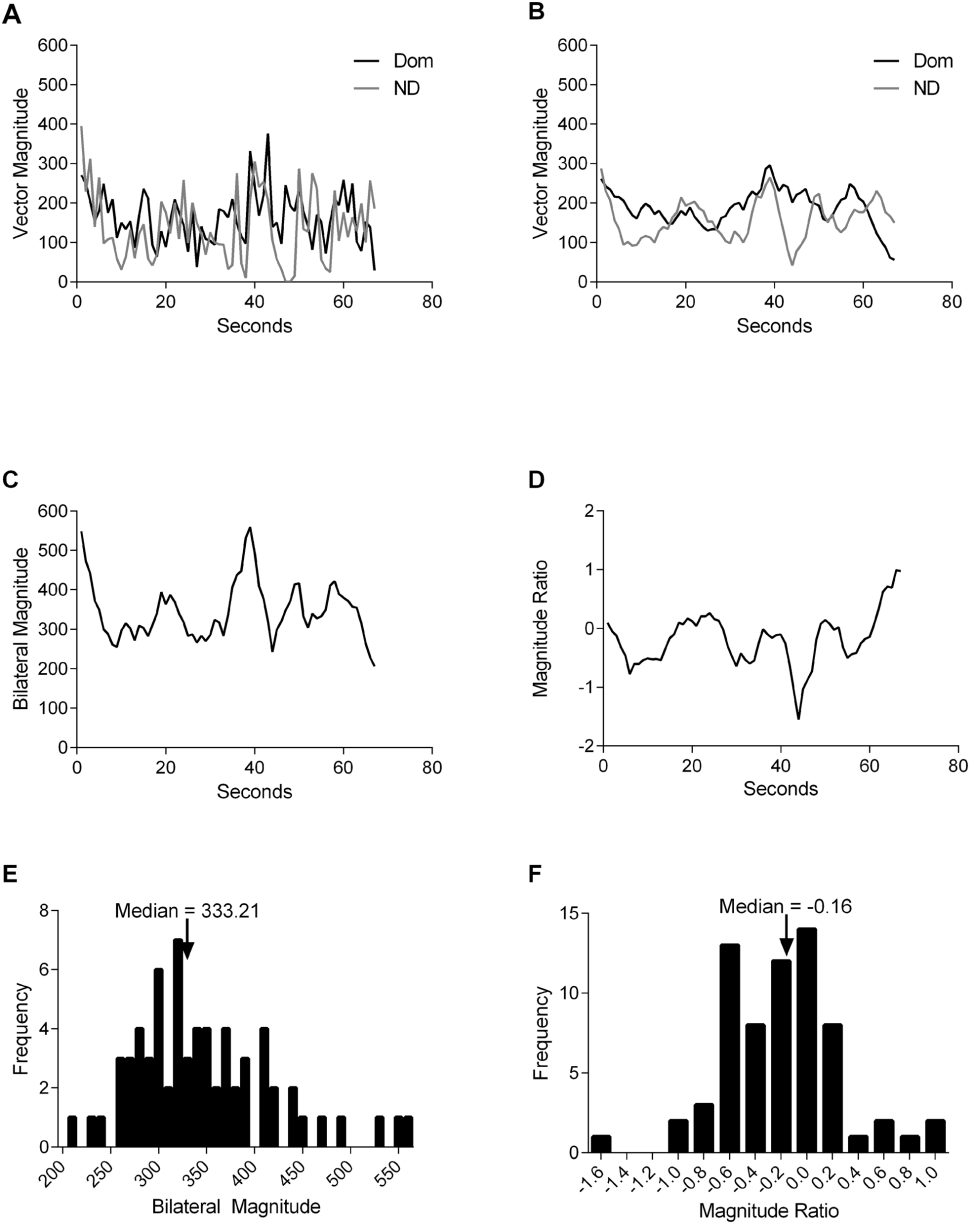
Example of data processing for one participant and one task, Grooming. A. Vector magnitude (measured in activity counts) for the dominant and nondominant UEs. B. Vector magnitudes were smoothed using a 5-sample moving average, resulting in decreased amplitudes. C. The Bilateral Magnitude (measured in activity counts) was calculated for each second of activity. D. The Magnitude Ratio was calculated for each second of activity. E & F. Histograms of Bilateral Magnitude and Magnitude Ratio values, respectively. The median values are identified by arrows.

Accelerometry data were used to calculate two primary variables of interest, the *Magnitude Ratio* and *Bilateral Magnitude*. Figure 1 illustrates how data were processed and primary variables calculated for one task to assist in explanation of the methods described below. Accelerometry data were exported from ActiLife 6 to MATLAB R2011b, and variables of interest were calculated using a custom-written program. First, for each second of data, activity counts across the three axes were combined into a single value, called a vector magnitude, using the equation: √(x^2^+y^2^+z^2^) (Fig. 1A) [8]. This was done separately for each UE. Second, vector magnitudes were smoothed using a 5-sample moving average to reduce the variability of vector magnitude amplitudes (Fig. 1B). Third, smoothed vector magnitudes were isolated for each task and were used to calculate the Bilateral Magnitudes and the Magnitude Ratios for each second of activity. We considered multiple options to quantify bilateral UE activity, but chose these primary variables because they most directly and intuitively reflected the constructs of interest, i.e. how the UEs are used together to accomplish tasks.

The Bilateral Magnitude reflects the intensity of activity across both UEs, and was calculated by summing the smoothed vector magnitude of the nondominant and dominant UEs for each second of activity (Fig. 1C). Bilateral Magnitude values of 0 indicate that no activity occurred, and increasing Bilateral Magnitude values indicate increasing intensity of bilateral UE activity.

The Magnitude Ratio reflects the ratio of acceleration between UEs. It was calculated for each second of activity by 1) adding one activity count to the smoothed vector magnitude of both UEs, 2) dividing the smoothed vector magnitude of the nondominant UE by the smoothed vector magnitude of the dominant UE, and 3) log-transforming the calculated values (Fig. 1D). The addition of one activity count was done to prevent dividing by zero for seconds when the dominant UE was inactive (i.e. denominator = 0). Log-transformation using a natural logarithm was performed to prevent positive skewness of untransformed ratio values greater than 1.0 [8]. Magnitude Ratio values of 0 indicate equivalent activity contribution from both UEs; positive values indicate more nondominant UE activity and negative values indicate more dominant UE activity, relative to the opposite extremity.

After calculating the Bilateral Magnitude and Magnitude Ratio for each second of each task, seconds when no activity in either extremity occurred (i.e. the Bilateral Magnitude was equal to zero) were removed for statistical analysis. Thus, only seconds when activity occurred in at least one extremity are reflected in the results. Seconds when no activity occurred were removed from statistical analysis because the purpose of this accelerometry-based methodology is to quantify bilateral UE activity *when UE activity occurs*, and inclusion of time when no activity occurred would influence statistical analyses.

In order to establish convergent validity of the primary variables, secondary variables were calculated that were expected to correlate with the primary variables. Secondary variables included *Estimated Energy Expenditure* and *Time Spent in Simultaneous Activity*. Estimated Energy Expenditure for each task was obtained from the 2011 Compendium of Physical Activities [16], which provides MET (Metabolic Equivalent of Task) values for various activities. One MET is defined as the amount of energy expended at rest, and equals 1.0 kcal*kg^−1^*h^−1^. MET values from 0–3 indicate light intensity activity, from 3–6 indicate moderate intensity activity, and above 6 indicates vigorous intensity activity [17], [18]. This secondary variable was expected to correlate with the Bilateral Magnitude.

Time Spent in Simultaneous Activity was defined as the percentage of time that both UEs were simultaneously active, and was calculated by dividing the number of seconds when the smoothed vector magnitudes of *both* UEs were simultaneously greater than 0 activity counts by the number of seconds when the smoothed vector magnitude of *either* UE was greater than 0 activity counts. Put more simply, Time Spent in Simultaneous Activity was calculated by dividing the number of seconds that both UEs were active by the number of seconds that at least one UE was active. Time Spent in Simultaneous Activity was expected to correlate with the Magnitude Ratio because these variables quantify bilateral UE activity in different, but related, ways (i.e. duration of simultaneous UE activity vs. ratio of acceleration between UEs).

In eight cases, few (n = 6) of the left-handed participants used their nondominant UE to complete tasks, even though all right-handed *and* half of the left-handed participants used their dominant UE to complete the same tasks. These cases are consistent with studies showing that left-handed adults complete some tasks with the nondominant UE more frequently than right-handed adults [19], [20]. For these eight cases, the inverse of the Magnitude Ratio values were used to correct for this inconsistency.

### Statistics

Statistical analyses were performed using IBM SPSS Statistics for Windows, Version 21 (IBM Corp., Armonk, NY). All variables at all stages of analysis were assessed for normality using Kolmogorov-Smirnov tests. Despite log transformation, all variables were not normally distributed; therefore, median values were calculated for participant- and sample-level analyses.

For each task and each participant, median Bilateral Magnitude (Fig. 1E) and median Magnitude Ratio (Fig. 1F) values were computed. Sample-level statistics were then calculated. For each task, the median and interquartile range of the median Bilateral Magnitude, median Magnitude Ratio, and Time Spent in Simultaneous Activity were computed. Outlying values were investigated but not removed because their effect on calculated median values was minimal.

Spearman correlation analyses were used to examine relationships between primary and secondary variables across all tasks. The correlation between the median Bilateral Magnitude and Estimated Energy Expenditure was examined using sample-level data because Estimated Energy Expenditure values were constant within tasks. The correlation between the median Magnitude Ratio and Time Spent in Simultaneous Activity was examined two ways: 1) using sample-level data for consistency with the approach used for the median Bilateral Magnitude and Estimated Energy Expenditure, and 2) using participant-level data to examine if the association was maintained across participants. We computed the median and interquartile range of the correlations coefficients across participants because the values were not normally distributed. Correlation coefficients 0.60 and higher were considered to be strong, between 0.30–0.59 were moderate, and 0.29 and lower were weak [21].

## Results

### Participants

Participants had a mean age of 54 (SD 11) years. Sex (female: n = 39/74) and race (African-American: n = 44/74, White: 30/74) were well-represented. The majority of participants were right-hand dominant (n = 62/74). Video-recordings of task performance were available for all but five typing tasks due to camera misplacement. No technical problems with the accelerometers occurred during the recording period.

### Analysis of primary and secondary variables

**Figure 2:**
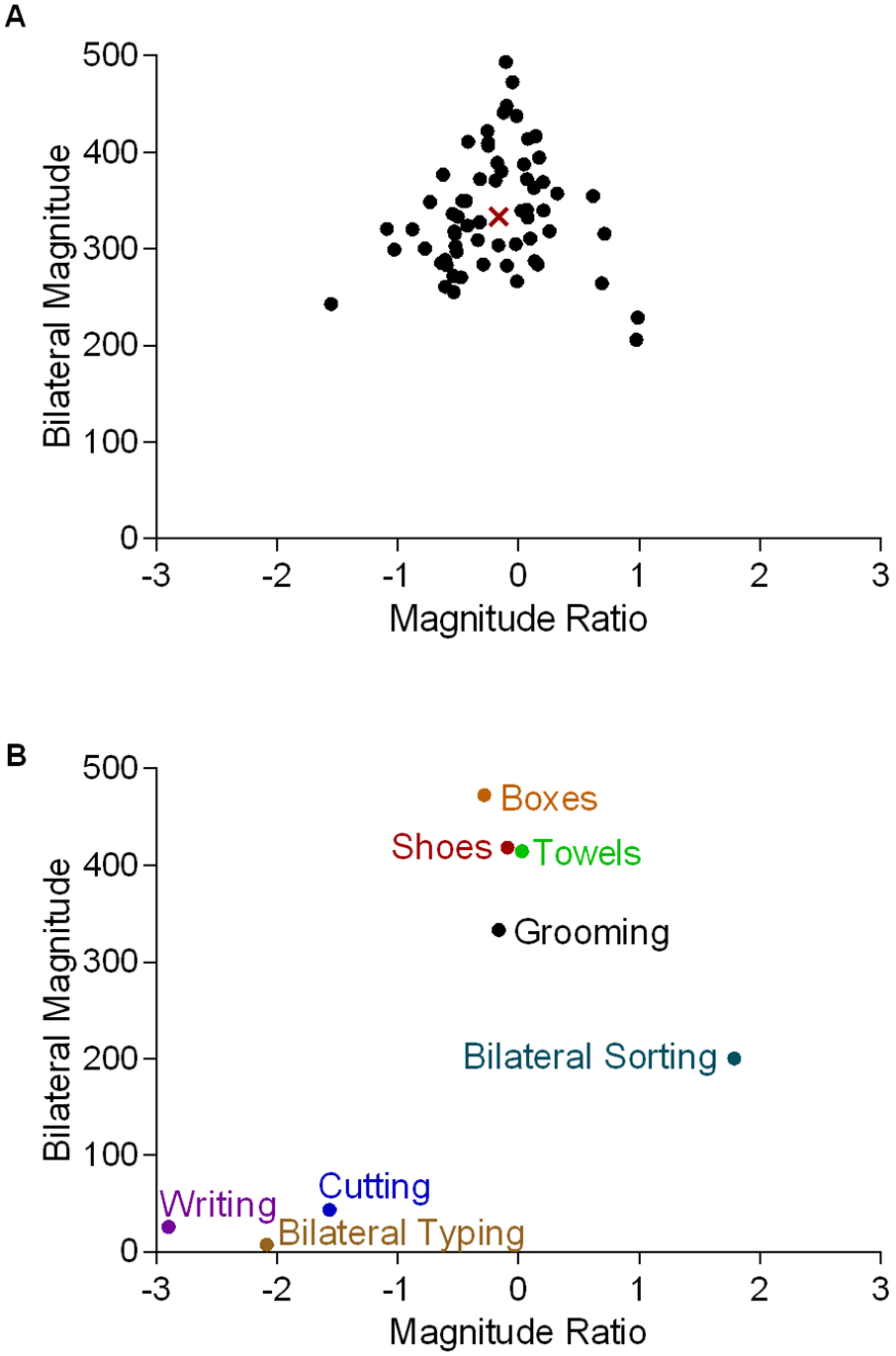
Example data for a single participant. A. Scatterplot illustrating the relationship between the Magnitude Ratio and Bilateral Magnitude (measured in activity counts) for each second of data (filled circles) for one task, Grooming. The median value of both variables is indicated by the red ‘X.’ B. Scatterplot illustrating how the different tasks compare to Grooming with respect to median Bilateral Magnitude and median Magnitude Ratio values. The median Magnitude Ratio for Bilateral Sorting and Bilateral Typing deviated from 0, despite these being bilateral tasks. For Bilateral Sorting, the participant used her nondominant UE to complete half of the task before using both UEs together. For Bilateral Typing, the participant frequently used her dominant UE to press the “Backspace” key, even though she used both UEs to type in a hunt-and-peck fashion.

Results for one participant, with a focus on a single task (Grooming), are presented first to facilitate understanding of sample-level data. The Magnitude Ratio and the Bilateral Magnitude both varied during the 70 seconds of task performance (Fig. 2A). Median values for each variable were calculated (see Fig. 1E, 1F, and 2A) to represent the bilateral UE activity of the task as a whole. Overall, this task was performed at a relatively high intensity (median Bilateral Magnitude = 333.21 activity counts), and the dominant UE was slightly more active than the nondominant UE (Magnitude Ratio = −0.16). Compared to Grooming, this participant performed some tasks more unilaterally as indicated by large, negative, median Magnitude Ratios (e.g. Writing & Cutting), and performed other tasks at both higher (e.g. Boxes) and lower (e.g. Cutting) intensities (Fig. 2B).

Median and interquartile range values of primary variables for all participants are presented in Table 2. Median Bilateral Magnitudes ranged from 5.63 to 463.36, indicating that the tasks were performed along a continuum of low to high bilateral UE intensity. Similarly, median Magnitude Ratio values ranged from −4.68 (Unilateral Sorting) where the dominant UE was used almost exclusively to complete the task, to 0.01 (Shoes & Towels) where both UEs contributed equivalently to task performance.

**Table 2:** Median and Interquartile Range of Median Bilateral Magnitudes and Median Magnitude Ratios for Each Task.

Activity (n)	Bilateral Magnitude†	Magnitude Ratio
	Median (IQR)	
Shoes (74)	281.32 (133.72)	0.01 (0.18)
Grooming (74)	309.69 (153.04)	−0.05 (0.28)
Boxes (74)	463.36 (78.27)	−0.05 (0.20)
Cutting (74)	50.39 (33.82)	−1.43 (1.19)
Towels (74)	426.60 (100.80)	0.01 (0.14)
Writing (74)	5.63 (6.56)	−1.95 (0.96)
Unilateral Sorting (38)	109.41 (30.19)	−4.68 (0.20)
Bilateral Sorting (36)	186.08 (183.06)	−0.14 (0.65)
Unilateral Typing (9)	19.09 (22.42)	−2.99 (1.50)
Bilateral Typing (60)	10.15 (12.58)	−0.39 (0.93)

n = number of observations for each task, see Methods.

† Unit of measurement  =  Activity Count.

Abbreviations: IQR, interquartile range.

**Figure 3:**
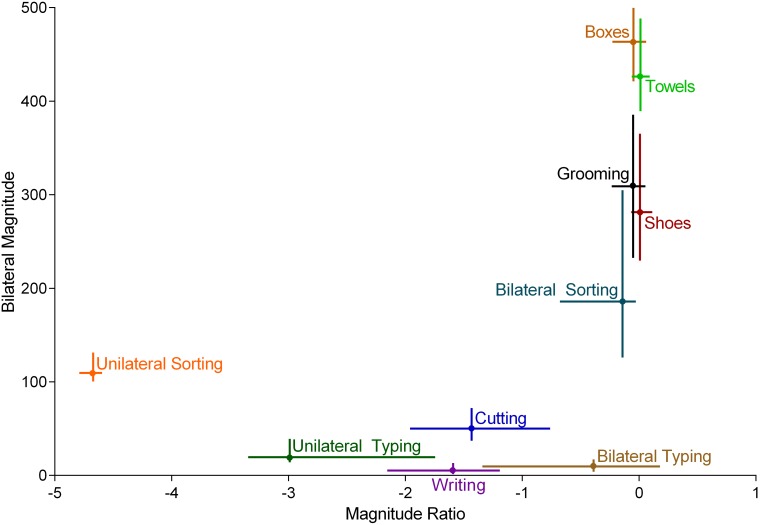
Sample data across all tasks. Values are the middle 50% (25–75 percentiles) of median Bilateral Magnitude (vertical bars, measured in activity counts) and median Magnitude Ratio (horizontal bars) values. Differences between tasks and variability within tasks are evident.

The middle 50 percent (25th to 75th percentiles) of median Bilateral Magnitude and median Magnitude Ratio values for each task across all participants are displayed in Figure 3. For the majority of tasks, median Bilateral Magnitudes and median Magnitude Ratios varied greatly across participants, indicating that the same task was performed very differently among individual participants. Despite the variability observed within tasks, tasks one might assume to be performed at higher intensities (e.g. Boxes) had high median Bilateral Magnitudes relative to tasks one might assume to be performed at lower intensities (e.g. Writing). Similarly, tasks one might assume to be performed with equal contribution from both UEs (e.g. Shoes) had median Magnitude Ratios near 0, while tasks that one might assume to be performed predominantly with the dominant hand (e.g. Unilateral Sorting) had large, negative, median Magnitude Ratios.

Values of secondary variables for each task across all participants are presented in Table 3. Estimated Energy Expenditure was low to moderate for the ten tasks. Nine out of ten tasks were categorized as light-intensity tasks (i.e. MET values less than 3), while one task (Boxes) was categorized as moderate intensity (MET values between 3 and 6). Both UEs were simultaneously active for a majority of tasks as indicated by a high percentage of Time Spent in Simultaneous Activity, while few tasks were completed relatively one-handed (e.g. Writing, Unilateral Sorting) as indicated by a low percentage.

**Table 3:** Values for Estimated Energy Expenditure and Time Spent in Simultaneous Activity for Each Task.

Activity (n)	Estimated Energy Expenditure†	Percent of Time Spent in Simultaneous Activity
		Median (IQR)
Shoes (74)	2.50	100.00 (0.00)
Grooming (74)	2.00	100.00 (0.00)
Boxes (74)	3.30	100.00 (0.00)
Cutting (74)	2.00	94.25 (21.72)
Towels (74)	2.00	100.00 (0.00)
Writing (74)	1.30	8.75 (16.11)
Unilateral Sorting (38)	2.50	8.89 (11.16)
Bilateral Sorting (36)	2.50	98.31 (32.03)
Unilateral Typing (9)	1.30	26.74 (35.61)
Bilateral Typing (60)	1.30	62.68 (47.47)

n = number of observations for each task, see Methods.

† As measured by MET values.

Abbreviations: IQR, interquartile range.

Spearman correlations were calculated between primary and secondary variables across all tasks. Estimated Energy Expenditure was strongly correlated with the median Bilateral Magnitude (ρ = 0.74, p<0.02). Time Spent in Simultaneous Activity was strongly correlated with the median Magnitude Ratio. This was true when correlations were examined using sample-level data (ρ = 0.93, p<0.01) and participant-level data (median ρ = 0.73, IQR = 0.28; correlation coefficients>0.71 were significant at p<0.05).

## Discussion

### General

The purpose of this study was to examine the validity of using the Bilateral Magnitude and Magnitude Ratio to quantify bilateral UE activity during the performance of everyday tasks. Visual inspection of Figure 3 provides face validity for the primary variables Bilateral Magnitude and Magnitude Ratio. Higher median Bilateral Magnitude values were observed for tasks where the UEs were used more intensively (e.g. Boxes, Towels) than when the UEs were used less intensely (e.g. Writing, Cutting). Median Magnitude Ratio values close to 0 occurred during tasks when both UEs contributed equally to task performance (e.g. Boxes, Towels), while large, negative Magnitude Ratios occurred during tasks when the dominant UE was predominantly used (e.g. Writing & Unilateral Sorting).

Strong correlations between primary and secondary variables were also demonstrated; that is, construct validity for the Bilateral Magnitude and Magnitude Ratio as metrics of real-world bilateral UE activity has been established. The strong correlation between median Bilateral Magnitudes and Estimated Energy Expenditure indicates that the Bilateral Magnitude is related to task intensity, which was expected given that activity intensity and activity magnitude are related measurements (i.e. intensity  =  magnitude per unit of time). The strong correlation between median Magnitude Ratios and Time Spent in Simultaneous Activity was also expected because both serve as indices of bilateral UE activity. The strong correlations between primary and secondary variables across tasks also indicate that the Bilateral Magnitude and the Magnitude Ratio quantify UE activity *independently* of the task performed. These data demonstrate validity of this methodology to quantify bilateral UE activity that occurs during the performance of everyday activity.

Methods that attempt to assess bilateral UE activity by calculating unilateral activity and then computing the ratio of activity between UEs provide an incomplete understanding of bilateral UE activity. For example, if both UEs are active for 12 hours each during a 24 hour period, the ratio of activity duration would be 1.0 (e.g. 12 hours/12 hours = 1.0). This value, however, could be obtained if both extremities were simultaneously active for 12 hours (i.e. bilateral activity), or if the extremities were unilaterally active for 12 hours each. In this situation, the ratio of activity duration does not provide accurate information about *bilateral* UE activity. Similarly, if the ratio of activity intensity during a 24 hour period were calculated, a similar situation would arise. In contrast, the methodology described in this study provides quantitative information on intensity of bilateral UE activity and the contribution of each UE to activity, *when activity occurs*. This is illustrated in Figure 2A, where one can appreciate that the intensity of bilateral UE activity and the contribution of each UE to activity *varies* over time.

Approaches that categorize UE activity using computer-based algorithms provide important information about UE activity, but not specifically about *bilateral* UE activity. Using accelerometry data, Schasfoort et al. [13] categorized UE activity into active and passive functional categories using multiple accelerometers placed on the thighs, trunk, and forearms with moderate to high accuracy. While data from both forearms was utilized by their algorithm to identify activity, no distinction was made between unilateral and bilateral activity.

Using a different approach, Bao & Intille [22] used five accelerometers placed on the ankle, thigh, hip, forearm, and upper arm to identify 20 *specific* UE tasks, including several performed exclusively with the UEs (e.g. scrubbing, eating). As in the previous example, bilateral activity was not distinguished from unilateral activity. Additionally, the algorithm was developed to identify only 20 tasks, which is a limiting factor because real-world activity consists of many more than 20 tasks. Furthermore, previous research [23], [24] has demonstrated that movement patterns across repetitions of the same task vary within individuals, which affects the accuracy of algorithms that are designed to identify specific tasks [25], [26]. Because movement patterns vary *within* individuals, one might also assume that movement patterns vary *across* individuals. Examination of the variability across participants for the median Bilateral Magnitude (see Figure 3), median Magnitude Ratio (see Figure 3), and Time Spent in Simultaneous Activity (see Table 3) confirms this assumption.

The methodology described in this study does not share the limitations outlined above because the Bilateral Magnitude and Magnitude Ratio quantify bilateral UE intensity and the contribution of each UE to activity when activity occurs, and is not limited to performance of specific tasks. Furthermore, only two accelerometers are needed to calculate the Bilateral Magnitude and Magnitude Ratio, which is an important consideration because wearing fewer accelerometers may improve wearing compliance in patient populations [27].

### Possible Applications

Analysis of UE activity using the Bilateral Magnitude and the Magnitude Ratio provides information about both the intensity of bilateral UE activity and relative contribution of each UE to activity performance. When the Bilateral Magnitude and Magnitude Ratio are calculated for known periods of time, such as during occupational or physical therapy treatment sessions, bilateral UE activity can be assessed within and across sessions to see if increases occur. Similarly, the Bilateral Magnitude and Magnitude Ratio can be calculated for activity that occurs outside of the clinic (e.g. while a patient is at home). The values can then be compared across time to see if increases occur. If increases do not occur, either across treatment sessions or across periods of real-world activity, a clinician may conclude that the treatment approach being used is not effective and that another one should be selected. Conversely, if values increase over time, evidence is provided that the treatment approach is effective in increasing UE activity. In this way, accelerometry-based measures of bilateral UE activity can be used in conjunction with clinical tests to assess recovery of UE function and real-world UE activity.

### Limitations

One limitation of this study is that small, observed finger movements in some participants may not have been recorded by the wrist-worn accelerometers, despite the established validity of accelerometers for measuring UE activity [8], [10], [11], [12], [13]. This potential underestimation of actual activity likely occurred because some hand movements can be made when the wrist and forearm are held still while the fingers move, as occurs in skilled typing. Many UE tasks, however, require coordinated movement of the fingers, hands, and forearm, as occurs when moving a computer mouse or reaching for and grasping a cup. This type of multi-joint activity will be captured by wrist-worn accelerometers. Additionally, the lack of recorded accelerometry data may have also resulted from the filtering algorithms utilized by the ActiLife software. If fine motor tasks are being studied, then the sensitivity of body-worn sensors and associated software for detecting small movements should be verified. This situation has a low probability of occurring in neurologic patient populations where large UE movements accompany fine-motor finger movements due to the inability to individuate joint movements [28].

A second limitation is that validation of the methodology described in this study is limited to tasks performed in a laboratory setting. This first stage of validation, however, is consistent with approaches used by other researchers. Both Uswatte [10] and Schasfoort [13] initially validated their methodologies using standardized laboratory tasks before applying their methodologies to real-world activity. Having demonstrated construct validity in this study, future studies will use the described methodology to examine real-world bilateral UE activity in healthy and patient populations. This will allow for comparison with existing accelerometry-based approaches (i.e. duration, intensity, and ratio of UE activity during a 24 hour period).

A final limitation is that participants performed sorting and typing tasks differently. Some tasks were performed unilaterally while others were performed bilaterally. Furthermore, participants performed bilateral tasks using a variety of symmetrical and complementary actions. In hindsight, this oversight was actually appropriate because in the real-world, the same task is performed differently within and across individuals. Importantly, the Magnitude Ratio was able to distinguish tasks performed using predominantly one extremity from those performed using both extremities.

## Conclusion

This study establishes the validity of an innovative methodology using accelerometry to assess bilateral UE activity during the performance of everyday tasks. The ability to quantify intensity of bilateral UE activity and the contribution of each UE to activity for real-world activity can be used by researchers and clinicians to select intervention approaches and evaluate the effectiveness of rehabilitation interventions. This is especially important in patient populations where bilateral UE function is impaired due to neurologic or orthopedic injury. Assessment of real-world bilateral UE activity can now be used in conjunction with clinical tests of function and patient-centered outcome measures to assess recovery of bilateral UE function in patient populations.
